# Efficacy of a Lactobacillus-Based Teat Spray on Udder Health in Lactating Dairy Cows

**DOI:** 10.3389/fvets.2020.584436

**Published:** 2020-10-23

**Authors:** John I. Alawneh, Ameh S. James, Nancy Phillips, Brandon Fraser, Karen Jury, Martin Soust, Timothy W. J. Olchowy

**Affiliations:** ^1^School of Veterinary Science, The University of Queensland, Gatton, QLD, Australia; ^2^Good Clinical Practice Research Group, The University of Queensland, Gatton, QLD, Australia; ^3^Terragen Biotech Pty Ltd., Coolum Beach, QLD, Australia; ^4^Faculty of Veterinary Medicine, University of Calgary, Calgary, AB, Canada

**Keywords:** teat end scores, lactobacillus-based, dairy cattle, mastitis, somatic cell counts, udder health

## Abstract

Teat disinfection is a common pre- and post-milking mastitis prevention practice that is part of a mastitis control program in dairy herds. Commercially available teat disinfectants are generally chemical-based products. The use of these products has occasionally raised concerns about the risk of chemical residues in milk. An alternative treatment or prevention strategy based on probiotics has the potential to circumvent this risk. Two treatments were compared in a cross-over clinical trial in a single herd: a lactobacillus-based, post-milking teat spray (LACT), and a commercial iodine-based post-milking teat disinfectant product as (positive control, PC). The effect of the two treatments on cow somatic cell counts was quantified using a multivariate mixed-effects linear regression model with cow fitted as a random effect. The odds of teat end scores increasing from a low to a high score tended to be lower (OR = 0.74, 95% CI 0.54–1.01, *P* = 0.06) for cows receiving LACT treatment. On average, there was also a tendency for a lower somatic cell counts in the LACT treated cows (antilog of coefficient = 0.91, 95% CI 0.80–1.03, *P* = 0.13) compared with the PC treated cows. The application of the lactobacillus-based product to teats could reduce the rate of teat end scores progression from low to higher scores, and potentially improve teat end sphincter functions and udder health. Further, larger scale validation work is required to support the findings of the current study.

## Introduction

Mastitis is the most prevalent production problem of animal welfare, production, and economic loss facing the dairy industry worldwide (1). The prevalence risk of mastitis is high (2, 3) and is influenced by animal (e.g., parity, stage of lactation), farm (e.g., herd-size, geographical location), and nutritional factors in the herd (1, 4). Teat canal and the integrity of teat-end tissue play a pivotal role against the introduction of mastitis-associated pathogens into the udder. Teat-end hyperkeratosis is the teat canal response to the forces imposed by milking. Milking machine and animal level factors can lead to severe teat-end hyperkeratosis and increase the roughness of the teat end (5, 6), and increase the risk of intra-mammary infections (IMI) by mastitis-causing pathogens in the herd (1, 6). Somatic cell counts (SCC) concentration in the milk is considered a biomarker of mammary gland inflammation and used as a proxy for IMIs (7, 8). A relative reduction in SCC while holding constant all other animal and herd-level risk factors, reflects a lower risk of exposure to IMIs (9). Reduced milk SCC is a reasonable indicator of effective mastitis management practices in the herd (10, 11).

Teat disinfection is a common mastitis prevention practice that has proven to be an excellent tool in the control of mastitis (12–14). This practice has been associated with a lower incidence of new IMI, a reduction in bulk milk SCC and fewer teat skin abnormalities (9). Ideally teat products have disinfectant properties and do not cause any harmful changes to the health of the teat skin [National Mastitis Council (NMC), www.nmconline.org]. Teat disinfectant formulations often include skin conditioning agents: emollients (lanolin) or humectants (glycerine, propylene glycol, or polyvinylpyrridone) (15). Some formulations contain aloe and allantoin which have been shown to have skin-healing properties (16). Teat disinfection that effectively and safely reduces bacterial load on teat skin reduces the risk of mastitis in the herd (17–19), improves teat skin condition (9), and reduces the risk of milk contamination (20). The observed efficacy of a teat disinfectant will vary depending on the production system, season, and the particular mastitis causing pathogens affecting the herd (18, 21, 22).

Gleeson *et al*. (17) conducted a study on two dairy farms in Ireland to explore udder health benefits of pre-milking teat disinfectant practice. In that study, bacterial numbers on teat skin were reduced and the practice was effective against environmental bacteria (*Escherichia coli* and *Streptococcus uberis*). Teat disinfection can also be a safe and effective method to reduce the incidence risk of mastitis caused by contagious pathogens such as *Staphylococcus aureus* (23, 24). However, it is less effective against environmental pathogens (15). A 2018 Australian study failed to demonstrate a benefit of iodine-based pre-milking teat disinfection (25). Treated multiparous animals had higher odds of clinical mastitis associated with environmental pathogens. Teat disinfection of primiparous animals did not reduce the odds of developing clinical mastitis compared to the untreated animals (Odd ratio [OR] = 1.31, 95% CI = 0.52–3.29). A combined pre- and post-milking teat disinfection program neither reduced the incidence of new IMI nor did it result in a reduction in SCC in a New Zealand dairy cattle study (9).

Commercially available teat disinfectants are generally chemical-based (iodophor, chlorhexidine) products (23). The use of these products has occasionally raised concerns about the risk of chemical residues in milk (26–28). Lactic acid bacteria (LAB) are part of the healthy alimentary microbiota (29), and have been proposed as a potential alternative therapy for the control of bovine mastitis (30, 31). A liquid product containing a mixture of *Lactobacillus* organisms (LACT) was developed as a post-milking teat spray. Therefore, the objective of the present study was to evaluate the short-term effect of LACT on mammary health as defined by SCC and teat end score (TES). It was hypothesized that LACT would be at least as effective as a commercially available iodine-based post-milking teat disinfectant in improving udder health.

## Materials and Methods

### Study Design

**Figure 1:**
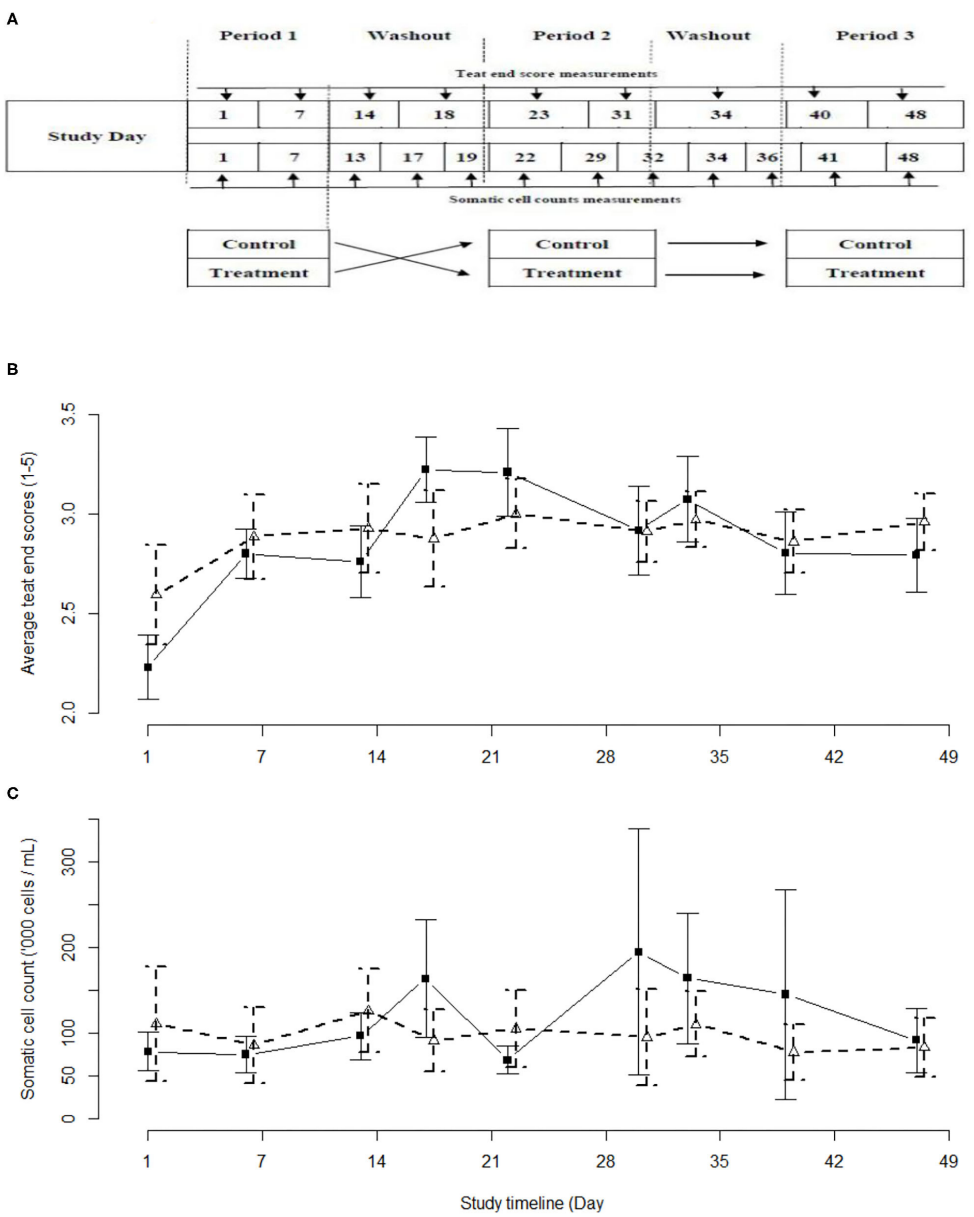
Schemata of the study design **(A)**, and the observed average teat end scores **(B)** and average somatic cell counts **(C)** with 95% confidence intervals as observed in the current study. Iodine-based (PC) group - black solid line and solid squares. Lactobacillus-based (LACT) group – black dashed line, white triangles. Treatment periods were between Study Days 1–7, 23–31, and 40–48. Washout periods were between study days 14–18, and 31–34.

This was a positive-controlled, randomized 2 × 3 cross-over study involving two experimental groups [LACT treated, positive control (PC) treated] and three treatment periods (Figure 1A). The study was conducted between 01 June and 26 July 2018 using the year-round calving University of Queensland-Gatton 230 milking cow research dairy herd. The herd is managed as two groups of milking cows typical of Queensland dairies: a combined fresh and early lactation group [up to 100 days in milk (DIM); fed a total mixed ration; *n* = 90]; and a second group comprising mid- and late lactation cows (>100 DIM; fed pasture and a mixed ration; *n* = 140). Grazing pasture consisted of a mixture of temperate and tropical plant species. Pasture supplemented with a silage-based mixed ration was sufficient to meet the maintenance and production requirements of a cow producing 25 L of milk per day. The experimental procedures were approved by the University of Queensland Animal Ethics Committee prior to the start of the study (Approval number: SVS/043/18/TERRAGEN).

The sample size required to evaluate changes in udder health (i.e., SCC), 13 cows, was based on the *a priori* assumptions for SCC of an alpha of 95%, a power of 90%, a common standard deviation of 20,000 cells/mL, a difference of 40,000 cells/mL and a correlation between group means of 0.1. For TES comparison, it was assumed that TES improvements (lower scores) would be associated with a decreased risk of mastitis in this herd. Sample size calculations determined that fifty cows would allow detection (with 95% confidence, power of 90%, pooled variance one teat end score, 1:1 ratio treatment to control sample size) of a difference of one teat end score (one to five scoring scale, see below) between the mean TES before and after LACT teat spray treatment. Therefore, the study sample size was 50 cows divided equally into the two experimental groups.

### Animal Management

Cows were milked twice daily at 04:00 and 15:00 h in a double 14 rapid exit high line parallel parlor (GEA Westfalia™, Victoria, Australia). The automatic cup removal (ACR) system was set to detach milking clusters when milk flow decreased to 0.2 L/min. Milk-line vacuum pressure was checked during the milking of each turn of cows in the parlor. The vacuum pressure was maintained between 45 and 48 kPa by a variable frequency vacuum pump. To ensure a consistent milking routine, all milking staff received ongoing training in milking machine operation and milking protocols. Based on study farm animal health records, the estimated incidence risk of clinical mastitis in the source herd was, on average, 22% [95% confidence interval (CI) = 17–33%].

### Teat End Scoring

The TES for each cow were determined by a single individual during a single milking session using the one to five TES scale adapted from Mein et al. (5): score 1 = normal with no apparent ring present at the teat end; score 2 = smooth and slightly rough ring; score 3 = rough ring; score 4 = very rough ring; score 5 = open lesions or scabs. The scorer was blinded to the treatment allocations to study animals. The TES were averaged (median) at the cow level. The median TES were used in subsequent analyses.

### Selection of Study Animals

Fifty apparently healthy lactating dairy cows of mixed age, stage of lactation, and breed, were randomly selected (simple random sampling without replacement) from a pool of 60 eligible cows (median TES of 2 or less, apparently normal quarter milk, SCC <300,000 for at least the past 8 weeks, no history of systemic disease or clinical mastitis in the 8 weeks preceding the study start date) (Supplementary Table 1). Enrolled cows were managed, fed, and milked as per the routine farm practices with the exception that the study animals were maintained as separate groups from the main herd.

### Experimental Design

The Lactobacillus based teat spray being evaluated was a proprietary liquid formulation (LACT; Lactolin™, Terragen Biotechnology Pty Ltd, Queensland, Australia) suitable for application as a post-milking teat spray. The preparation consisted of a mixture of three *Lactobacillus* spp. (*Lactobacillus paracasei, L. buchneri, L. casei*; minimum 10^6^ cfu/mL of each strain) in saline (0.9%NaCl). This preparation was stored at 4°C until applied to the teats (without an emollient) using a hip mounted, hand operated mobile teat sprayer (HipSpray™, Ambic Equipment Ltd., Davies Way, Brisbane, Australia) set to deliver 10 mL per teat as per manufacturer instructions. The positive control treatment (PC) was a commercial, iodine-based, post-milking teat disinfectant (Dairy Power Mastidyne™ Iodophor 20 g/L available iodine, 2% free iodine, ECOLAB, Sydney, Australia) supplied as a 2-part concentrate and automatically mixed with potable water on an as needed basis for application to the teats using a spray wand. The product as applied consisted of three parts Dairy Power Mastidyne™ Iodophor teat sanitiser, eight parts cool potable water, and one-part Dairy Power Glysoft™ udder emollient 10% solution (ECOLAB, Sydney, Australia) as per the manufacturer instructions. The inline sprayer gun (AMBIC™, Davisway/DASCO, Victoria, Australia) was set to pump a volume of 10 mL per teat covering the entire teat as per manufacturer instructions.

Cows were assigned to groups using simple random sampling without replacement. Simple random sampling was also used to assign a specific teat treatment (either LACT or PC) to one of the two 25-cow study groups for the first and second 2-week long experimental periods. The third 2-week long experimental period was a replica of the second period (Figure 1A). The two groups of animals were milked separately with an abbreviated milking machine cleaning cycle run before and between the milkings. To minimize the risk of residual effect of the two treatments, each treatment period was separated by a minimum washout period of 48 h during which there were no applications of either treatments. This minimum washout period was based on an absence of detectable (qPCR) biological residues of the LACT organisms at 36 h post-treatment.

Composite milk samples (50 mL) were collected bi-weekly from each cow at the Monday and Friday morning milkings, preserved with Acticide L-Bronopol, and SCC determined by the Australian Herd Recording Services (Kenilworth, Queensland, Australia) using an automated cell counter (Fossomatic 5000, Foss Electric). Teat ends were evaluated and scored weekly.

### Statistical Analysis

Summary statistics generated for continuous or categorical variables (as appropriate) included: mean, median, standard deviation (SD), first and third quartiles (1st and 3rd Q), minimum, maximum, counts and percentage, as appropriate. *Chi-squared* test was used to assess the homogeneity of TES count distribution between the treatment groups. The statistical analysis was conducted in R (43).

The association between the LACT and PC groups and TES was assessed using a multivariate, mixed effects, ordered logistic regression model. The analysis was performed at the quarter-level to allow the model to account for the repeated measurements in TES and clustering of teats within cows (the sampling unit, i.e., the udder quarters nested within the cow, i.e., the experimental unit). Square root transformation was applied to the study day to maintain the linearity of the modeled log odds across the study days and reduce the risk of violating the proportional odds assumption (see below). Statistical significance was declared at an alpha of 0.05 or less. The association between LACT and PC and individual cow SCC was quantified using a multivariate mixed-effects linear regression model with cow fitted as a random effect. Model building followed forward selection procedure. First order interaction terms were tested and were retained if interaction terms was significant at a likelihood ratio test *P* value of 0.05 or less. Models specification followed that described by St-Pierre (44) and took the following generic forms:

**Somatic cell counts – animal level data [1]**

$$\begin{array}{l} y_{ijk}=Intercept+Treatment_{i}+Period_{j}+\beta_{1}Time+S_{ik}+\epsilon_{ijk} \end{array}$$

**Teat end scores – quarter level data [2]**

$$\begin{array}{l} y_{ijmk}=Intercept+Tretament_{i}+Period_{j}+\beta_{1}Time\\ +Quarter_{m}+S_{mik}+\epsilon_{ijmk} \end{array}$$

Where *y*_*ijk*_ and *y*_*ijmk*_ denote the response observed at the cow and quarter levels, respectively for model [1] and [2], in period_*j*_ of treatment_*i*_, and Si_*k*_ and *S*_*imk*_ are the random error term for the *k*^th^ cow (or *Quarter*_*m*_ nested within cow, respectively) in the *i*^th^ treatment group. The outcome variable (SCC) was log transformed to stabilize the SCC variance and restore normality of the data. Study day was modeled as a polynomial (of the 4th order) variable with the order of the polynomial determined using the Akaike Information Criterion (AIC). The residuals (ε_*ijk*_ *and ε*_*ijmk*_) of the random effect term were assumed to be normally distributed with a mean of zero, a variance of σ^2^, and an autoregressive correlation structure of the first order. Overall model fit was based on AIC, Bayesian information criterion (BIC) and visual assessment of *Pearson's* residuals against fitted values, *Q-Q* standardized residuals against standardized normal quantiles violated the normality assumption (32). The proportional odds assumption was checked visually by examining the vertical consistency of distances between any two of the orders TES scores (at the logit scale) within explanatory variable in the model. The overall effect of LACT and PC over the three treatment periods was further explored using least-squares means (LSM) prediction from the final mixed-effects linear model. (LSM means predictions were averaged predictions across all covariates in the model fixed at the reference levels). Statistical significance was declared at an alpha of 0.05 or less. Because SCC were log transformed, interpretation of the coefficient represents a unit change in log SCC. The antilog of each coefficient is interpreted as follows: for a continuous explanatory variable, the antilog of the coefficient represent a change in average (geometric mean) SCC for each unit change in the continuous variable. For a categorical variable, the antilog of the coefficient is the ratio of the means for each level of the categorical variable compared with the reference category. All analysis using ordinal (33), emmenas (34), visreg (35), nlme and lme4 (32, 36) statistical packages in R.

## Results

The teat end scores of the cows in the LACT and PC groups followed a similar curvilinear trend (Figure 1B). The TES values were highest during the second of the three 2-week treatment periods. Overall, the LACT group was associated with fewer TES 4 (13%) and 5 (1%) and more TES 1 (7%), (χ^2^_29.042, df = 4_, *P* < 0.01) compared to the PC group (15, 2, and 2%, respectively, Figure 1B and Supplementary Table 2). In parallel with the TES results, the SCC associated with both treatments followed a similar trend (Figure 1C).

The results from the multivariable model for TES are shown in Table 1. Holding the covariates at their reference, on average, the odds of a shift from low to high TES tended to be lower for cows in the LACT group compared to TES for cows in the PC group (OR = 0.74, 95% CI 0.54–1.01, *P* = 0.06; Table 1). The TES value of individual cows at baseline influenced the odds of observing a high TES during the study. Irrespective of treatment assignment, for each unit TES score above score 1 at baseline, the odds of TES changing from a low to a high score during the study increased ~3-fold (OR = 3.46, 95% 2.07–5.78, *P* < 0.01; Table 1).

**Table 1:** Coefficient (standard errors) and odd ratios (95% confidence interval) from final multivariate mixed-effects ordered logistic regression model fitted on cows teat end scores (TES^†^) for the study animals.

**Variable**	**Coefficient (SE)**	**Odd ratio (95% CI)**	***P*-value**
Teat end scores at baseline	1.24 (0.26)	3.46 (2.07–5.78)	<0.01
Daily milk production	−0.01 (0.02)	0.99 (0.95–1.03)	0.59
(L; centered^‡^)			
Time (Day; square root)	−0.01 (0.01)	0.99 (0.98–1.01)	0.15
Treatment group			
PC	**Reference**	1	
LACT	−0.30 (0.16)	0.74 (0.54–1.01)	0.06
Treatment period			
Period 1	**Reference**	1	
Period 2	2.03 (0.18)	7.61 (5.36–10.80)	<0.01
Period 3	1.95 (0.29)	7.01 (3.98–12.39)	<0.01
Udder quarter			
Fore quarters	**Reference**	1	
Hind quarters	−1.32 (0.19)	0.27 (0.18–0.39)	<0.01
Random effect	Variance (SE)	95% Confidence Interval	
Cow	0.33 (0.18)	0.11–0.94	
Quarter	0.60 (0.20)	0.31–1.15	

† *Teat end scores (scale 1–5; one is a normal teat end with no ring apparent; 5 is a severely abnormal teat end, rough, raised, and obvious ring at teat end)*.

‡ *Centered on the mean*.

*SE, Standard Error; CI, Confidence Interval; L, liters; PC, iodine-based positive control; LACT, lactobacillus based product. Model fitted using mixed-effect linear regression procedure in R. Model fitted with quarter nested with cow and animal fitted as random effect. Robust standard error estimation was used to adjust for clustering within cow. Final model AIC = 2700.561, Wald Chi-squared = 237.20 P <0.001, Log psuedolikelihood = −1337.2804*.

The results from the multivariable model for SCC are shown in Table 2. After controlling for the effect of TES at baseline, milk production, and holding the remaining covariates at their reference, on average, there was a tendency for SCC in the LACT group to be 9% lower (antilog of coefficient = 0.91, 95% CI 0.80–1.03, *P* = 0.13) compared to the PC group (Table 2). Across all three treatment periods, the average SCC for cows in the PC group was 14% higher (1.14, 95% CI = 0.97–1.33, *P* = 0.07; (Table 3 and Supplementary Figure 1) compared to the LACT treated cows.

**Table 2:** Coefficient (standard errors) and antilog of the estimated coefficients (95% confidence interval) from final multivariate mixed-effects linear regression model fitted on individual cows somatic cell counts (SCC; 000's cells/mL) for the study animals.

**Variable**	**Coefficient (SE)**	**Antilog of coefficient**	***P*-value**
		**(95% CI)**	
Intercept	4.38 (0.18)	82 (56.03–119.99)	<0.01
Log SCC at baseline	0.81 (0.08)	2.24 (1.91–2.61)	<0.01
(centered^†^)			
Study day (4th order polynomial^‡^)			
1st order	4.38 (2.19)	79.55 (1.07–5922.20)	0.04
2nd order	−1.13 (0.95)	0.32 (0.05–2.05)	0.23
3rd order	−1.31 (0.75)	0.27 (0.06–1.17)	0.08
4th order	1.56 (0.74)	4.77 (1.12–20.40)	0.04
Treatment group			
PC	**Reference**	–	
LACT	−0.09 (0.06)	0.91 (0.80–1.03)	0.13
Treatment period			
Period 1	**Reference**	–	
Period 2	−0.37 (0.21)	0.69 (0.45–1.04)	0.07
Period 3	−0.53 (0.30)	0.59 (0.32–1.05)	0.07
Random effect	Variance (SE)	95% Confidence Interval	
Cow	0.52 (0.02)	0.48–0.56	

† *Centered around the mean*.

‡ *The order of the fitted polynomial was assessed using model AIC*.

*Se, Standard Error; CI, Confidence Interval; PC, iodine-based positive control; LACT, lactobacillus-based product. Model fitted using mixed-effect linear regression procedure in R. Model fitted with cow fitted as random effects. Final model AIC = 628.929, Loglikelihood = −303.4645*.

**Table 3:** Least-square means predictions (marginal means; at the log and antilog scales) and mean ratios obtained over the grid of predictors settings from linear mixed-effects model shown in Table 2.

**Experimental group**	**Predicted marginal effect**				
	**Log scale**		**Antilog scale**		
	**Mean (SE)**	**95% CI**	**Mean (SE)^†^**	**95% CI^†^**	***P*-value^‡^**
PC (Iodine-based	4.62 (0.13)	4.35–4.89	102 (14)	75–138	
Positive control)					
LACT (Lactobacillus-based)	4.50 (0.13)	4.23–4.76	90 (12)	66–121	
PC / LACT means ratio					
	0.13 (0.07)	0.08–0.94	1.14 (0.08)	0.97–1.33	0.07

† *Values are in 000's cells/mL'*.

‡ *Bonferroni adjusted for multiple comparisons*.

*SE, Standard Error; CI, Confidence Interval*.

## Discussion

Our study evaluated the effect of a probiotic-based, post-milking teat skin spray on the health of the mammary glands using the proxy parameters of SCC and TES. This short-term crossover designed pilot study does support the hypothesis that the probiotic product was at least as effective as a commercial iodine-based post-milking teat disinfectant product. There were fewer abnormal TES in the LACT group. The odds of a TES shifting (throughout the study) from a lower to a higher score was lower for cows in the LACT group. Even though there was no statistically significant differences in the SCC values in response to the two treatments, when the effect of the explanatory variables was controlled, there was a trend to a lower mean SCC in the probiotic group.

The study did not identify any significant difference in TES between LACT and PC groups. Teat end scores for cows receiving either treatment followed a similar curvilinear trend. The odds of a reduction in the average TES did not differ between groups. There was a relatively increased number of chapped teat ends in the control group cows. This occurred despite the skin conditioning properties of this commercial product relative to the product under study. The conditioner and sanitizer components were mixed and used throughout the study as per manufacturer recommendations. The mixing system for the commercial product was serviced regularly by a qualified milking machine technician to ensure correct operation. However, no iodine analyses were performed on the final product as delivered to the cows. In contrast to treatment with the commercial product, cows receiving the probiotic product showed a strong propensity toward a lower risk of an increase in TES. This suggests the *Lactobacillus*-based product has a protective effect on teat ends. The pattern of change in SCC (tendency for reduced SCC in the LACT group) in this study is consistent with the reduction in subclinical mastitis in association with the use of a *Lactobacilli* spp. based teat treatment observed by Yu et al. (20). They suggested that the effect of the biologic-based treatment was to decrease the exposure of the teat to mastitis-associated bacteria by improving the microbial environment of the cow teat. The current study focused on the effect on milk SCC and did not investigate the potential effect on the microbiota of the teat.

The specific mechanisms responsible for the observed beneficial trends associated with the *Lactobacillus*-based product were not investigated in the present study, but several possibilities exist. If the organisms in the LACT group grow faster than mastitis causing pathogens, there may be fewer sites on the teat skin for pathogens to adhere or colonize thereby reducing the exposure risk to the udder (19, 37). The development of bovine mastitis has been associated with dysbiosis, an imbalance between the healthy microbiota of the mammary gland and mastitis causing pathogens (37). It is possible that the presence of the LACT organisms inhibited the development of any teat skin or mammary gland dysbiosis. The use of probiotics to minimize the risk of (or correct existing) dysbiosis has been proposed as a method to both reduce mastitis risk and the need for antimicrobial use (29). An additional potential protective effect may be the result of barrier-like biofilm properties of the organisms (20). *Lactobacillus* spp. have some characteristics needed for biofilm formation. They do colonize and are retained for long periods, a critical factor in preventing colonization by pathogenic bacteria (38). The role of the established resident microbiota of the teat skin and mammary gland and the potential changes to the microbiota in response to treatment were not investigated. Changes in TES and SCC in response to potential pathogen exposure were not investigated by culture or PCR based examination of epithelial surfaces or milk samples. This is a limitation of the current study and should be addressed in future and larger scale study. Therefore, the results presented in the current study should be interpreted with caution.

The product tested in this study contained live *Lactobacilli* organisms. This type of product offers advantages over those which do not contain living organisms (i.e., lactic acid, iodine, and other chemical products) as several mechanisms are in force: bacterial competition and/or displacement from an ecological niche(s), and production of anti-bacterial substances [bacteriocins such as lacticin (39)]. This product's characteristics may result in an ongoing effect in contrast to the one-time high dose exposure associated with commercial chemical based teat disinfectants; a high initial dose which may taper off below the threshold of efficacy. In addition, this type of product would benefit from the lack of harmful residues, a characteristic associated with GRAS (“generally regarded as safe”) organisms. The future role of lactobacillus-based udder health products is most likely as externally acting formulations. Intra-mammary infusion of these GRAS organisms has been associated with increases in SCC of the infused quarter, especially if the initial SCC of the quarter was quite low (40). Similar outcomes have been observed by others (15, 41). This effect was not observed in quarters with a pre-existing high SCC (IMI affected quarters). Cure rate of infected high SCC quarters following administration of the probiotic product (Lacto-bac; *Lactobacillus acidophilus, Lactobacillus casei*) was inferior to the antimicrobial treated group of cows. The authors did not provide any *in vitro* antibacterial test results so it is possible that these organisms were either not producing, or were producing insufficient amounts of bacteriocins.

One of the challenges of field studies is the inability to control factors that may have significant impact on the study outcomes. This study was no exception with the deterioration of weather conditions (dry, cold); and the lack of treatment of application in the washout phase, during the second treatment period that may have influenced the observed outcomes. With a few exceptions, dairy cows in Australia and New Zealand are not housed in barns. Outside of the time spent in a milking parlor (more often than not with open side walls), under shade structures (purpose-built and/or trees), or sheltering behind wind breaks, the cows are subjected to the weather conditions of the pasture/paddock. The cows in this study had access to partial protection offered by fixed sun shade structures and a modest wind break created by the side of a building. Dry cold weather conditions during the second treatment period were associated with higher SCC values and TES in both groups. The effect was greater in the PC group, but not statistically significant (Figure 1). Martins et al. (42) reported a similar increase in SCC and TES and described an increased growth rate of mastitis pathogens during the winter season. It is possible this lack of significant group differences in response to adverse weather was influenced by the sample size of the present study. In cold temperate climates, it is not uncommon for producers to use a “winter formulation” teat disinfectant to reduce the “teat end chapping effect” of the weather and reduce the risk of intra-mammary infection. Further comparative formulation studies would be required to test this potential effect and evaluate the value of a “winter formulation” under Australian conditions.

By their nature, preliminary, pilot or proof-of-concept type studies have limitations. This study is no exception with limitations being expected. The sample size may have been too small to detect significant differences in treatment effects. The short duration and relatively restricted exposure of cows to seasonal variations may have hidden potential long-term beneficial effects of the treatments. The purpose of the study was to test a hypothesis and determine if any potential benefit existed to support the conduct of a long term multiple season (hot and humid, cool, and dry) study. As the observational data from this pilot study was limited, it was not possible to determine if beneficial effects may develop over a longer term, such as a complete lactation, or when cows in both experimental groups are allowed to comingle (e.g., shared risk of exposure mastitis causing pathogens, homogenous animal management within groups). No cows developed any adverse reactions or illnesses, either local (mastitis) or systemic, during the course of the study supporting the general acceptance that this probiotic product is indeed appropriately categorized as GRAS when used in this manner. Finally, determination of the mechanisms underlying any beneficial effects require further laboratory and field studies. Enough evidence was acquired to encourage further investigation of the interaction of these organisms with the teat and udder microbiota.

## Conclusions

Somatic cell counts followed a similar trend for cows receiving either lactobacillus based LACT product or iodine-based PC product. Overall, cows in the LACT group had fewer teat end scores of one, four, and five. The odds of an increase in the teat end scores and average somatic cell counts over the three treatment periods tended to be lower for cows treated with lactobacillus based product compared with the iodine-based PC treated cows. The results from this pilot study suggest that lactobacillus-based product treatment could improve teat end sphincter functions and udder health. Further, larger scale validation work is required to support the findings of the current study.

## Data Availability Statement

The raw data supporting the conclusions of this article will be made available by the authors, without undue reservation.

## Ethics Statement

The animal study was reviewed and approved by the University of Queensland Animal Ethics Committee.

## Author Contributions

JA study design, sample collection, sample processing, data analyses, and drafting of the manuscript. AJ data interpretation and drafting of the manuscript. NP and BF data collection. MS and KJ supply of LACT product and LACT product quality control. TO study design, data interpretation, and drafting of the manuscript. All authors contributed to the article and approved the submitted version.

## Conflict of Interest

KJ and MS were employed by the company Terragen Biotech Pty Ltd. The remaining authors declare that the research was conducted in the absence of any commercial or financial relationships that could be construed as a potential conflict of interest.
