# Supplementary material for: Efficacy of a Lactobacillus-Based Teat Spray on Udder Health in Lactating Dairy Cows
Source: Front Vet Sci. 2020 Oct 23;7:584436. doi: 10.3389/fvets.2020.584436 (PMC7644449; doi:10.3389/fvets.2020.584436)
Supplement: Supplementary file 1 [file Data_Sheet_1.docx]

**Supplementary Table 1.** Descriptive statistics of lactation and animal attributes at baseline (study day -7 to study day -1) by experimental group (i.e. groups have not been assigned to a specific treatment).

|  | **Experimental Group** | | | | | | | | **P^†^ Values Group 1 *vs.* Group 2** |
| --- | --- | --- | --- | --- | --- | --- | --- | --- | --- |
| **Treatment Period** | **Group 1 (n=25)** | | | | **Group 2 (n=25)** | | | |  |
|  | ***n* (%)** | **Mean (SD)** | **Median (Q1, Q3)** | **Min, Max** | ***n* (%)** | **Mean (SD)** | **Median (Q1, Q3)** | **Min, Max** |  |
| Current lactation total milk (litres) |  | 3476 (1940) | 2820 (2216, 3558) | 1428, 8028 |  | 3983 (2254) | 2939 (2495, 5382) | 1861, 11290 | 0.39 |
| Average daily milk (litres) |  | 25 (7) | 23 (20, 29) | 16, 46 |  | 23 (6) | 23 (19, 27) | 12, 35 | 0.23 |
| Parity |  | 2 (2) | 1 (1, 3) | 1, 7 |  | 2 (1) | 2 (1, 2) | 1, 5 | 0.92 |
| Days in milk (days) |  | 137 (74) | 112 (90, 141) | 50 ,323 |  | 162 (93) | 128 (97, 254) | 75 ,424 | 0.29 |
| Teat end scores |  | 1.81 (0.70) | 2 (1, 2) | 1, 4 |  | 2.05 (0.89) | 2 (1, 3) | 1, 5 | 0.81 |
| Somatic cell count (×1000 cells/mL) | | 77 (58) | 61 (40, 90) | 20, 264 |  | 84 (97) | 66 (19, 87) | 7, 387 | 0.81 |
| Distribution of observed teat end scores | |  |  |  |  |  |  |  | 0.79**^‡^** |
| Score 1 | 44 (0.35) |  |  |  | 34 (0.28) |  |  |  |  |
| Score 2 | 62 (0.50) |  |  |  | 55 (0.46) |  |  |  |  |
| Score 3 | 18 (0.14) |  |  |  | 24 (0.20) |  |  |  |  |
| Score 4 | 1 (0.01) |  |  |  | 5 (0.04) |  |  |  |  |
| Score 5 | 0 (0.00) |  |  |  | 2 (0.02) |  |  |  |  |
| Breed |  |  |  |  |  |  |  |  | 1.00**^§^** |
| Friesian or Friesian cross | 13 (52) |  |  |  | 12 (48) |  |  |  |  |
| Crossbreed | 12 (48) |  |  |  | 13 (52) |  |  |  |  |

† P values derived from One Way Analysis of Variance (ANOVA) procedure with exception of teat end score distribution and breed.

‡ Kruskal-Wallis ꭕ^2^ _0.072258, df = 1_; P-value = 0.7881

§ *Pearsons’* ꭕ^2^_0, df = 1_ with *Yates'* continuity correction; P-value = 1

**Supplementary Table 2:** Summary statistics of teat end scores^†^ stratified by treatment period, study day and experimental group.

|  |  | **Experimental Group** | | | | | |
| --- | --- | --- | --- | --- | --- | --- | --- |
| **Treatment Period** | **Study Day** | **PC (Iodine-based Positive control)** | | | **LACT (Lactobacillus-based)** | | |
|  |  | **Mean (SD)** | **Median (Q1, Q3)** | **Min, Max** | **Mean (SD)** | **Median (Q1, Q3)** | **Min, Max** |
| Period 1 | 1 | 2.23 (0.72) | 2 (2, 3) | 1, 4 | 2.59 (0.85) | 2 (2, 3) | 1, 5 |
|  | 7 | 2.80 (0.72) | 3 (2, 3) | 1, 4 | 2.89 (0.75) | 3 (2, 3) | 1, 4 |
| Washout | 14 | 2.76 (0.67) | 3 (2, 3) | 2, 4 | 2.93 (0.78) | 3 (2, 3) | 1, 5 |
|  | 18 | 3.22 (0.61) | 3 (3, 4) | 2, 4 | 2.88 (0.81) | 3 (2, 3) | 1, 5 |
| Period 2 | 23 | 3.21 (0.66) | 3 (3, 4) | 2, 5 | 3.00 (0.65) | 3 (3, 3) | 2, 5 |
|  | 31 | 2.92 (0.74) | 3 (2, 3) | 2, 5 | 2.91 (0.64) | 3 (3, 3) | 2, 5 |
| Washout | 34 | 3.07 (0.77) | 3 (3, 3) | 2, 5 | 2.97 (0.63) | 3 (3, 3) | 2, 5 |
| Period 3 | 40 | 2.80 (0.75) | 3 (2, 3) | 2, 5 | 2.86 (0.57) | 3 (3, 3) | 2, 4 |
|  | 48 | 2.79 (0.68) | 3 (2, 3) | 2, 5 | 2.96 (0.60) | 3 (3, 3) | 2, 5 |

† Teat end scores (scale 1 to 5; one is a normal teat end with no ring apparent; 5 is an abnormal teat end, rough, raised and obvious ring at teat end)


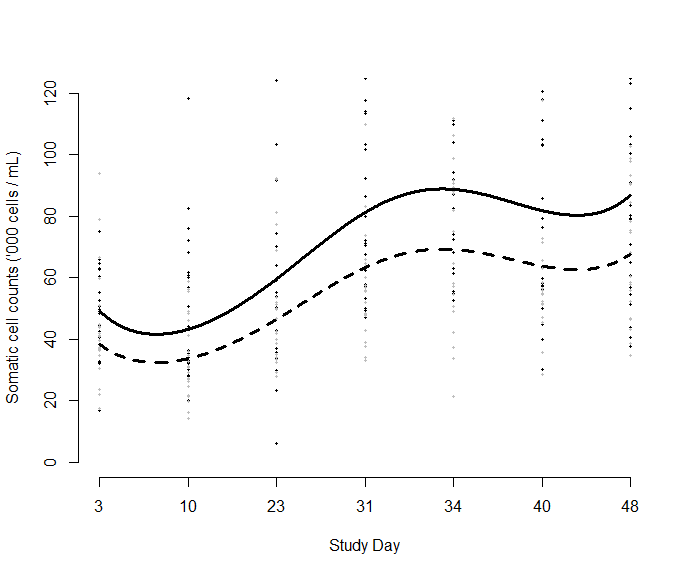


**Supplementary Figure 1**. Line plot of best linear unbiased average somatic cell count predictions for PC (Iodine-based Positive control; solid line, solid black diamonds) and LACT (Lactobacillus-based; dashed line, solid grey diamonds) as estimated from the linear mixed effects model in Table 2.
